# Morpho-Functional Alterations in the Gills of a Seawater Teleost, the Ornate Wrasse (*Thalassoma pavo* L.), after Short-Term Exposure to Chlorpyrifos

**DOI:** 10.3390/toxics8040097

**Published:** 2020-11-07

**Authors:** Rachele Macirella, Vittoria Curcio, Elvira Brunelli

**Affiliations:** Department of Biology, Ecology and Earth Science, University of Calabria, Via P. Bucci 4/B, 87036 Rende (Cosenza), Italy; rachele.macirella@unical.it (R.M.); vittoria.curcio@unical.it (V.C.)

**Keywords:** organophosphorus pesticides, seawater fish, gills morphology, ultrastructure, confocal microscopy, Na^+^/K^+^-ATPase

## Abstract

Chlorpyrifos (CPF) is an organophosphorus insecticide commonly used for domestic and agricultural purposes. The risk posed by environmental contamination from CPF is well acknowledged, and it has been detected worldwide in aquatic habitats and coastal areas. In addition, due to its slower degradation in seawater compared to freshwater, CPF is of particular concern for marine environments. Here, we investigated for the first time the morpho-functional alterations induced by CPF on the gills of *Thalassoma pavo*, a widespread species in the Mediterranean Sea. We tested the effects of two sublethal concentrations (4 and 8 µg/L) after 48 and 96 h. Our study demonstrates that the alterations induced by CPF are dose and time-dependent and highlight the harmful properties of this insecticide. After exposure to the low tested concentration, the more frequent alteration is an intense proliferation of the primary epithelium, whereas after exposure to the high concentration, the primary epithelium proliferation is less extensive, and the most evident effects are the thinning of secondary lamellae and the ectopia of chloride and goblet cells. CPF also modulated the expression of Na^+^/K^+^-ATPase. Dilation of lamellar apical tips, pillar cell degeneration, and appearance of aneurysms are often observed.

## 1. Introduction

Human population growth has led to the overexploitation of lands for agricultural purposes resulting in a proportional increase in global use of pesticides and fertilizers [1]. Considering the world population should reach around 9 billion people by 2050 [2], it is unlikely that pesticide use will be reduced in the near future, with detrimental consequences for public and environmental health [3]. Organophosphorus compounds (OPs), due to their high efficiency and less persistence in the environment, have been employed in agricultural applications since the late nineteenth century as a viable alternative to organochlorines [4]. Currently, OPs are the most widely used pesticides, accounting for almost 40% of the global market, and their application is still growing [5,6,7]. The extensive and intensive use of OPs has led to increased contamination of aquatic habitats, which appear to be one of the primary locations for OPs [8,9].

Chlorpyrifos (CPF), patented and introduced on the global market in 1965 [10], is a broad-spectrum OP and one of the most widespread pesticides in freshwater ecosystems. It has been widely detected in fishery products, both farmed and wild, worldwide [7,9,11,12], and for its high bioaccumulation tendency, pseudo persistence, and toxic characteristics, it has been categorized as a priority substance for the protection of aquatic ecosystems within the European Commission Water Framework Directive [13]. Moreover, human epidemiological studies demonstrated that occupational exposure to chlorpyrifos induces severe pathological effects, raising considerable concern for exposure by means of fish consumption [14]. In January 2020, the European Commission formally decided not to renew the authorization and withdrawal of CPF starting from the spring; however, in the U.S., the CPF registration is up for renewal in 2022 [15]. Moreover CPF is one of the most commonly used pesticides in developing areas [7] and a selling increase is expected for the next few years in consideration of a growing global market demand [6]. Hence, while CPF is banned in several countries and will be dismissed in others in the next few years, like the U.S., it is still being used in some developing areas where it could pose a risk to wildlife.

In aquatic environments, CPF pollution may result from direct or unintended wastewater discharge from agricultural, urban, or industrial activities or through rainfall, runoff, and air-drift. The CPF concentration in surface water peaks during the periods of seasonal application on crops, thus evidencing the considerable agriculture contribution to CPF pollution [16]. CPF enters the sea through coastal areas and rivers, and it has been detected all over the word in both water and sediment [8,16]. Of particular concern is the long seawater persistence of CPF [17], indicating a slower degradation than in freshwater. In the Mediterranean Sea, CPF concentrations are highly variable, ranging from a few ng/L (4.8) to 303.8 µg/L [16,18,19].

For decades, marine ecosystems have been subjected to several anthropogenic pressures, and at a global level, no area has been unaffected [20,21]. Chemical pollution is among the most important pressures affecting marine areas, and the extent of the impact has been greater in coastal zones compared to offshore areas. Since marine coastal regions have a high ecological and economic value and provide a wide diversity of ecosystem services, a dedicated action plan is crucial to reduce hazards and environmental risks [20]. In 2010, the Marine Strategy Framework Directive (MSFD) [22] implemented the legislative drivers of European countries, according to the Convention on Biological Diversity (CBD) [23]. Following maritime legislation, monitoring the health of the ecosystem, and improving knowledge on the effects of marine biodiversity stressors are required to promote conservation and sustainable development [20]. Despite this, few laboratory studies have examined the effects of CPF in seawater organisms, and the consequences of CPF exposure in marine fish are largely unknown. Since teleost fish are the most abundant group of vertebrates on the planet, they have been widely used as bioindicators in most aquatic ecosystems and are ever more acknowledged as good indicators of marine pollution [24].

It is widely recognized in fish that CPF can be absorbed through the digestive tract and permeable tissues and bioaccumulate in different organs, including gills [12,25]. The two better-characterized mechanisms of action by which CPF exerts its toxicity are acetylcholinesterase inactivation and oxidative stress induction [26,27]. However, chlorpyrifos elicits a number of other harmful effects interfering with steroid receptors and thyroid hormones, thus acting as an endocrine-disrupting chemical (EDC), and it is also able to alter fish behavior and locomotion [28,29].

The fish gills play an important role in several physiological functions such as gas exchange, osmoregulation, and excretion, and are widely used in ecotoxicological studies as a biomarker of environmental pollution. Due to their wide surface area and direct contact with external medium, gills are one of the organs most affected by xenobiotics, and histopathological investigations of this organ are therefore considered a sensitive tool for fish health assessment [27].

Research on CPF has mainly focused on freshwater species, and severe histological alterations have been reported in the gill apparatus after long-term exposure [25,30,31,32]. Surprisingly only two studies investigated the morphological responses of the gills in seawater fish, and available information deal with CFP effects after 30 days of exposure in coastal and estuarine fingerlings species [33,34].

Morphological changes of target organs usually reflect contaminant levels in the aquatic medium and time of exposure [35] and are often used to aid in interpreting toxicological data.

This study does not present information on the toxic properties of CPF that could be better obtained through other research approaches. Here, we show adverse morphological effects elicited by CPF in the gill apparatus of a marine Teleost in a field-found application scenario, which for OPs corresponds to short-term exposure as they are employed through repeated short applications.

Although the morphological alterations *per se* cannot explain the mechanism of action of toxic substances, they could help understand toxicity pathways that underpin toxic effects.

Here, we investigate, for the first time, the effects of short-term exposure to CPF on the ornate wrasse *Thalassoma pavo*, a widespread marine species of the Mediterranean Sea. The morphological and ultrastructural alterations of gills induced by two concentrations of CPF (4 and 8 µg/L) have been evaluated after 48 and 96 h of exposure. Moreover, considering that the osmoregulatory impairment of gills function has been suggested as one of the toxic mechanisms of CPF, we also examined the expression of the Na^+^/K^+^-ATPase, a sensitive biomarker in xenobiotics-induced osmotic stress [36,37]. This study will contribute to the evaluation of the risk related to CPF presence in coastal marine ecosystems.

## 2. Materials and Methods

### 2.1. Fish Maintenance

Specimens of *T. pavo* (48 healthy adults of both sexes with a mean body mass of 9.48 ± 0.45 g) were captured by baited traps in coastal waters near San Lucido, a location on the Tyrrhenian coast of Southern Italy. Animals were transported in the laboratory for the acclimatization period (2 weeks) and kept in 80 L aquaria (no more than 15 fishes per tank). Aquaria were equipped with filter and oxygenation systems and filled with seawater from the sampling location. The concentrations of nitrite and nitrate, the absence of pollutants, and the water parameters of each aquarium were constantly monitored (salinity = 35%, density = 1.027–1.028 g/cm^3^, temperature = 18–24 °C, dissolved oxygen at 8.0–8.6 mg/L, 100 mg hardness CO_3_Ca/L); animals were fed every 2 days with commercial fish food (Tetramin) and maintained under a natural light/dark cycle (12/12 h). 

### 2.2. Experimental Design

Dose selection was made considering the environmental contamination from CPF worldwide and in the Mediterranean Sea [16,38]; therefore, two concentrations (4 and 8 μg/L) were used for the present investigation. A range-finding test was performed, confirming that both concentrations are sublethal (unpublished data). The two nominal concentrations were prepared by dissolving CPF (purity of 99.5%, Chem Service Inc, West Chester, PA, USA) in an organic solvent (i.e., acetone) and adding it to the treatment tanks filled with seawater.

The experiments were carried out using a semi-static acute experimental set-up, with the renewal of the solution every 24 h, following the standard procedure [39]. Water samples for chemical analysis were collected at the beginning and within 12 h of the renewal of test solutions. Actual chlorpyrifos concentrations were verified via Gas Chromatography with Flame Photometric Detector (Agilent 6890 N; Agilent Technologies, Santa Clara, CA, USA). 

For each experimental unit (10 L glass tanks), four fishes of comparable body dimensions were exposed to the two selected CPF concentrations. The control group (*n* = 8) was maintained in untreated seawater (10 L glass tanks) with the equivalent amount of organic solvent used for CPF dilution (100 µL of acetone; less than 0.001%). Two replicates were done for each experimental unit, including the control. During the experiment, water quality parameters (i.e., pH, temperature, hardness, salinity, density, dissolved oxygen, and photoperiod) were monitored daily and maintained as reported above. Fish were fed everyday ad libitum with commercial fish food; debris and food waste were removed using a fine-meshed mesh.

At the two selected time points, 48 and 96 h, fish were anesthetized and euthanized using sulfated methane tricaine (MS 222, Sandoz, Sigma-Aldrich, St. Louis, MO, USA). Excised gills from each experimental unit, including the control, were processed for light, electron, and confocal microscopy.

Animal care, experiments, and killing have been undertaken according to the European Convention for the protection of vertebrate animals used for experimental and other scientific purposes (Council of Europe No. 123, Strasbourg, 1985) and the Italian regulation (DL 116, 27 January 1992), which do not require any explicit authorization by an ethics committee for the used species.

### 2.3. Light and Transmission Electron Microscopy

Gills were fixed for 4 h by immersion in 4% glutaraldehyde (Electron Microscopy Sciences, Hatfield, PA, USA) in phosphate-buffered saline (PBS 0.1 M, pH 7.2, 4 °C) and post-fixed for 2 h with osmium tetroxide (1% in PBS). After dehydration in graded ethanol, samples were placed in propylene oxide and embedded in Epon-Araldite (Araldite 502/Embed 812, Electron Microscopy Sciences, Hatfield, PA, USA). Samples were cut Using a Leica UltraCut UCT (Leica Microsystems, Wetzlar, Germany); semi-thin sections (1µm thickness) were stained with toluidine blue and observed using a LM Leitz Dialux 20 EB (Leica Microsystems, Wetzlar, Germany). Ultra-thin sections (800Å thickness) were stained with a uranyl acetate replacement, contrasted with lead citrate (Electron Microscopy Sciences, Hatfield, PA, USA), and finally observed under a Zeiss EM 10 electron microscope (Zeiss, Oberkochen, Germany).

### 2.4. Immunohistochemistry

Samples for immunohistochemical analyses were fixed for 48 h by direct immersion in Bouin’s fluid, in phosphate-buffered saline (PBS, pH 7.1), dehydrated in an increasing series of ethanol, cleared in xylene, and embedded in paraffin wax (mean fusion point = 56 °C). Using a rotary microtome Leica RM 2125 RT (Leica Microsystems, Wetzlar, Germany), gill samples were cut (10 μm thickness) and mounted on positively charged slides. Deparaffinized sections were subjected to the indirect immunofluorescence technique. After several washes in 0.1 PBS, sections were first incubated at room temperature with 20% normal goat serum (30 min) and finally incubated at 4 °C (overnight) with a mouse monoclonal anti Na^+^/K^+^-ATPase (Developmental Studies Hybridoma Bank, Iowa City, IA, USA; working dilutions of 1:100).

The next day, after several washes in PBS, sections were incubated at room temperature in the dark (30 min) with the second antiserum, a fluorescein isothiocyanate-conjugated γ-globulin goat anti-mouse (Sigma-Aldrich Chemical Co., St. Louis, MO, USA; working dilutions of 1:50). On washed slides (0.1 PBS), propidium iodide (Sigma-Aldrich Chemical Co., St. Louis, MO, USA; working dilutions of 1:200), which binds to ribonucleic acid and labels cell nuclei, was applied. Slides were washed again in PBS and finally mounted and observed under a Leica TCS SP2 Confocal Laser Scanning Microscope (Leica Microsystems, Wetzlar, Germany).

### 2.5. Quantification and Statistical Analyses

Semi-thin sections, stained with toluidine blue, were used for both qualitative and semiquantitative histological analyses. First, slides of each animal from all experimental groups, including the control, were observed under an LM Leitz Dialux 20 EB (Leica Microsystems, Wetzlar, Germany) and scored by the dichotomous parameter presence or absence of lesions. Fisher’s exact probability test at the significance level of 0.001 was used for statistical validation.

At a later time, histological alterations were evaluated semi-quantitatively. Each slide has been observed at different magnifications (20×, 40×, 100×) to unveil any alterations. The degree and extent of alterations were assessed using the appropriately modified grading system of Georgieva and colleagues [40]. The grading system was: (−) no histological alterations; (+/−)-mild histopathological alterations; (+) moderate histopathological alterations; (++) severe histopathological alterations; (+++) very severe histopathological alterations of the gill surface architecture.

To compare CPF exposed groups to the control, with respect to the Na^+^/K^+^-ATPase expression, immunolabelled slides coming from a subset of fish (8 from the control and 8 from each CPF-exposed groups) were randomly chosen, observed, and finally photographed by a Leica TCS SP2 confocal microscope. The area of positively expressing cells belonging to a field of 875 × 875 pixels (350 × 350 μm) was measured using an image analysis software (NIH, developed at the National Institutes of Health; Bethesda, MD, USA) and the values, indicating the area expressing the fluorescent signal in each section, have been compared using the two-way ANOVA followed by Bonferroni’s multiple comparison tests (at a significance level of 0.01). Data were checked for normality and homogeneity of variances (Kolmogorov–Smirnov test for normality and Bartlett for homogeneity) and presented as mean ± standard deviation of the mean. We used GraphPad Prism 2007 version 5.00 for Windows (GraphPad Software, Inc., San Diego, CA, USA) to run all statistical analyses.

## 3. Results

CPF exposure resulted in several histopathological alterations that could be observed in all individuals from both exposed groups, and the extent of the damages was dose and time-dependent (Table 1; *p* < 0.001).

The summary of the histological alterations found in each experimental group and the respective grade of severity is reported in Table 2.

### 3.1. Histology and Ultrastructure

#### 3.1.1. Control Group

The morphology and ultrastructure of *T. pavo* gill apparatus under basal conditions have already been described in detail [41], and only a brief description will be furnished in this paper.

**Figure 1:**
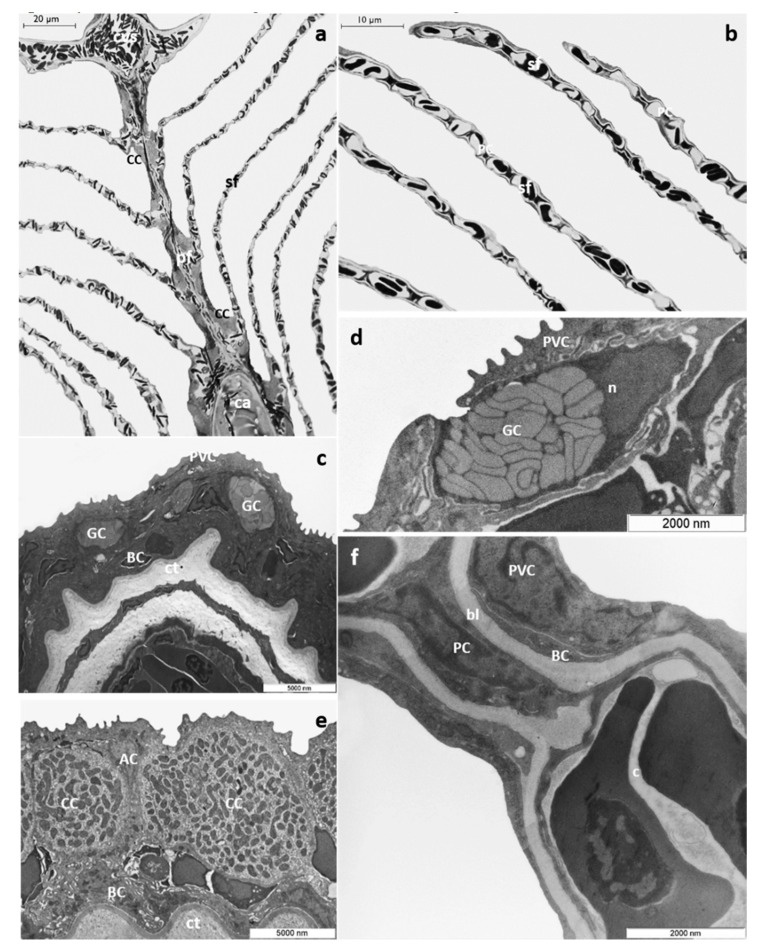
*T. pavo* gills under basal conditions. (**a**) Light micrograph of a primary gill filament (pf) and secondary lamellae (sf). Note the chloride cells (CC) distributed along the filament margins and in the interlamellar region; ca=cartilage, cvs=central venous sinus. (**b**) Light micrograph of secondary lamellae (sf); PC= pillar cell. (**c**) Ultrastructural organization of the primary epithelium; PVC= pavement cell, GC= goblet cell, bc= basal cell; ct= connective tissue. Note the PVCs in the outermost layer. (**d**) High magnification micrograph of a goblet cell (GC) showing large clear electron granules; PVC= pavement cell, n= nucleus. (**e**) High magnification micrograph of a chloride cell (CC); note the numerous mitochondria and the typical tubular vesicular system; AC= accessory cell, BC= basal cell, ct= connective tissue. (**f**) TEM micrograph showing a secondary lamella; PVC= pavement cell, BC= basal cell, PC= pillar cell, bl= basal lamina.

*T. pavo* gills show the general arrangement typical of marine Teleosts; each gill is supported by four pairs of gill arches that give insertion to a double series of primary filaments (i.e., main filament). From each side of the primary filaments, secondary lamellae depart perpendicularly (Figure 1a,b). A multilayer epithelium covers the main filament (primary epithelium) comprising four cells types: pavement cells (PVCs), which represent the most abundant cell type, undifferentiated basal cell (BCs), mucous cells or goblet cells (GCs), and chloride cells (CCs), which in marine Teleosts are associated with accessory cells (ACs) (Figure 1c–e). Mucous cells exhibit a round or oval shape and are characterized by large clear electron granules that occupy the entire cytoplasm (Figure 1d). CCs are mainly distributed in the interlamellar region of the main filament and show a typical apical crypt. Their cytoplasm is filled with numerous mitochondria associated with a complex ramified tubular vesicular system (Figure 1e). BCs, lying on the connective tissue, form the inner epithelial layer (Figure 1c–e).

The lamellar epithelium (secondary epithelium) comprises two layers: an external layer made by PVCs and an inner layer made by undifferentiated BCs. The pillar cells (PCs) support and define the capillary blood flow throughout the lamellae (Figure 1f).

#### 3.1.2. CPF Exposed Group, Low Tested Concentration: 4 µg/L

**Figure 2:**
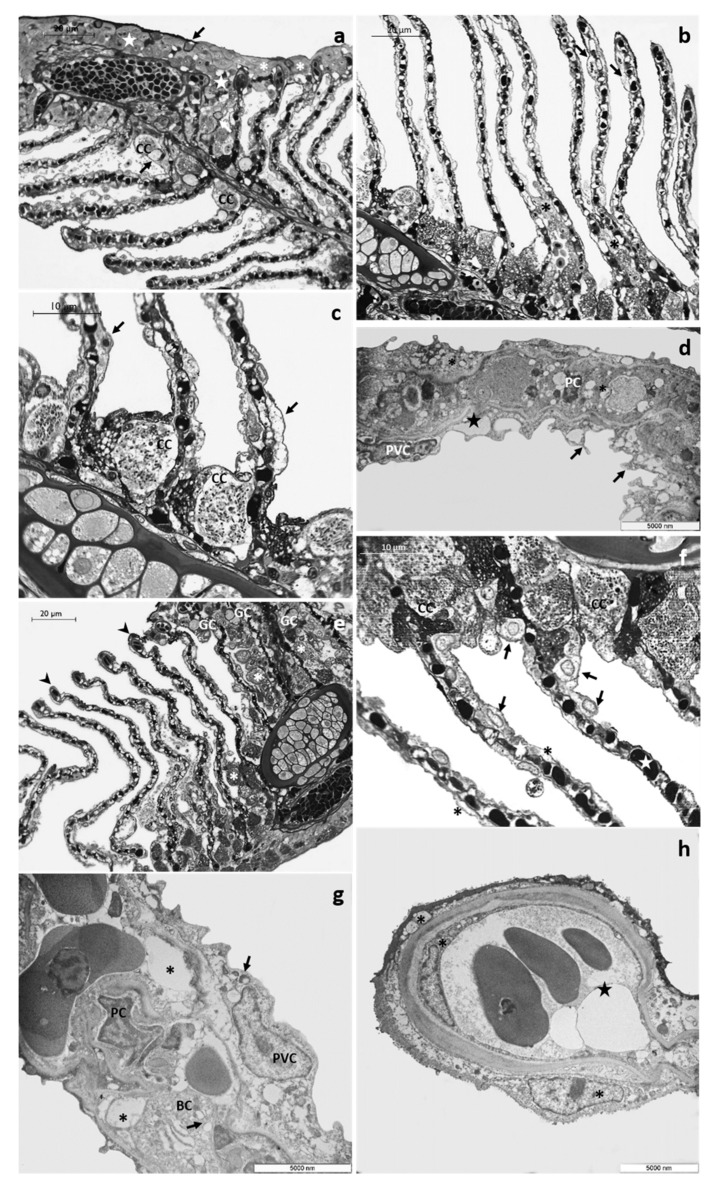
Representative micrographs of *T. pavo* gills after 48 (**a**–**d**) and 96 (**e**–**h**) hours of exposure to 4 µg/L of CPF. (**a**) Light micrograph showing the proliferation of primary epithelium (star) and the fusion of the lamellae in their apical portion (asterisks). Note the presence of goblet cells (black arrows) in the proliferative region of the main filament and in the interlamellar space; CC = chloride cell. (**b**) Note the main epithelium extending throughout the interlamellar area (asterisks) and lacunae’s appearance in the secondary epithelium (black arrows). (**c**) Detail of chloride cells (CC) characterized by a poor cytoplasm; also note the lacunae in the secondary filament (black arrows). (**d**) TEM micrograph showing cytoplasmic projections originating from pavement cells (black arrows) and the highly vacuolated cytoplasm of both epithelial and endothelial cells (asterisks); star = lacunae, PVC = pavement cell, PC = pillar cell. (**e**) Light micrograph showing the proliferation of primary epithelium (asterisks) and the increase in the number of goblet cells (GC). GCs are also detected in the proliferative areas; note the appearance of aneurysms in the lamellar apical tips (black arrowheads). (**f**) The lifting of the secondary epithelium (asterisks) and degenerated cell characterized by a scant cytoplasm, are more easily recognizable with further enlargement. Note in the proximal portion of the lamellae chloride cells distributed in clusters (CC) and in the secondary lamellae the degeneration of the vascular system (stars). (**g**) TEM micrograph showing the poor cytoplasm of epithelial cells (black arrows) and the wide intercellular lacunae (asterisks); PVC = pavement cell, BC = basal cell, PC = pillar cell. (**h**) Details of both modified epithelial and endothelial cells (asterisks); star = dilated apical tips.

After 48 h of exposure to the low tested concentration, it is possible to observe the appearance of fusion areas among adjacent lamellae, particularly evident in their distal end, and the proliferation of primary epithelium (Figure 2a). Numerous small goblet cells are scattered throughout the main epithelium and extend into the proliferative region between adjacent lamellae (Figure 2a). These cells could also be recognized in the interlamellar space and are often associated with CCs (Figure 2a). In the secondary filaments, the detachment of the external layer from the underlying connective tissue is an extensive process involving almost all the respiratory lamellae (Figure 2b). This proliferative phenomenon is better appreciated with further magnification, which also reveals the appearance of wide spaces and lacunae and the poor cytoplasm of some CCs (Figure 2c).

Ultrastructural observations show the presence of long cytoplasmic projections arising from PVCs that give the epithelial surface an irregular appearance. Moreover, the lifting of external cells originates wide intra-epithelial lacunae, and both epithelial and endothelial cells present a highly vacuolated cytoplasm (Figure 2d).

Both structural and ultrastructural alterations increase after 96 h of exposure. The proliferation of primary epithelium is evident, leading to the obliteration of interlamellar space. In the primary epithelium, the number of mucous cells further increases, and it is possible to observe numerous GC also in the newly formed epithelium between adjacent lamellae (Figure 2e). In the distal portion of lamellae, it is possible to note aneurysms’ formation (Figure 2e). The lifting of secondary epithelium is more extensive, and intensive and swollen degenerating cells are often seen (Figure 2f). These cells could be easily distinguished by their pale cytoplasm and are mainly distributed at the epithelial surface of secondary filaments (Figure 2f). Numerous CCs, sometimes hypertrophic, are distributed in clusters in the lamellae proximal portion (Figure 2e,f). Some CCs maintain their typical features, whereas others display a scant cytoplasm (Figure 2f). The vascular component is also strongly compromised, and the PCs system is disorganized (Figure 2f). Under TEM, it is possible to recognize the poor cytoplasm of epithelial cells and the appearance of wide intercellular spaces and lacunae (Figure 2g). In the dilated apical tips, degenerating epithelial cells could be detected along with vacuolization of endothelial cells cytoplasm (Figure 2h).

#### 3.1.3. CPF Exposed Group, High Tested Concentration: 8 µg/L

**Figure 3:**
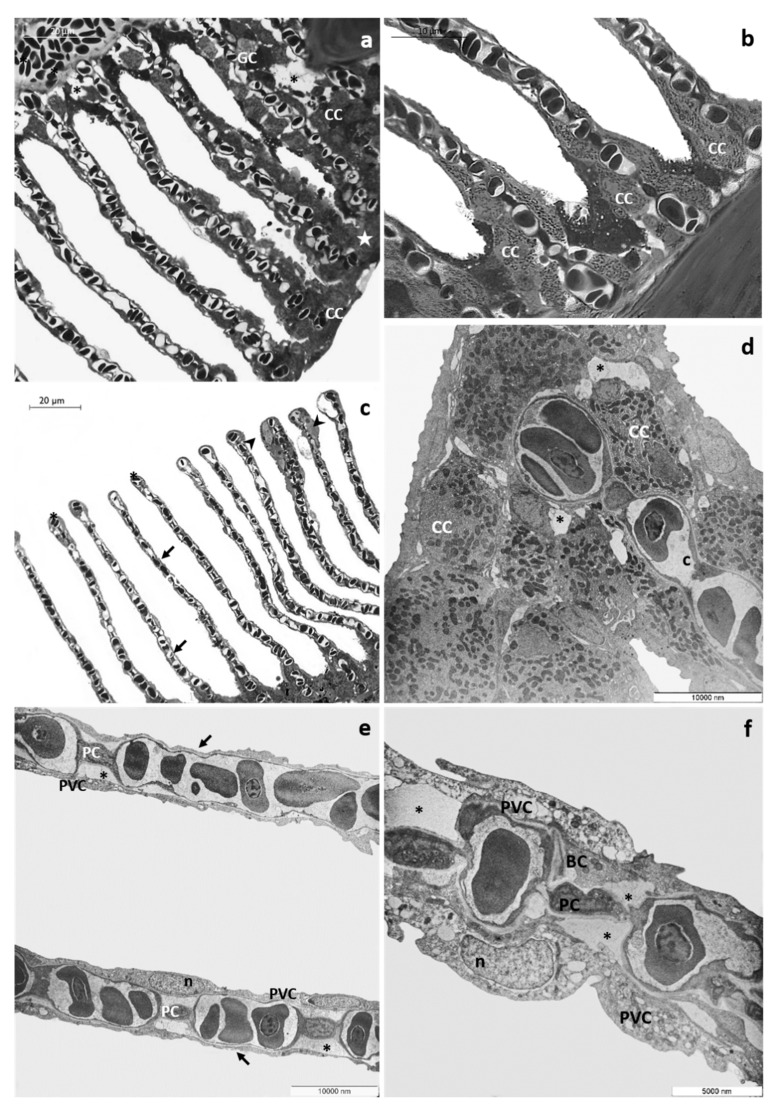
Representative micrographs of T. pavo gills after 48 h of exposure to 8 µg/L of CPF. (**a**) Light micrograph showing the proliferation of primary epithelium (star). The detachment of the primary epithelium from the underlying connective tissue (asterisks) could also be observed. Note the ectopia of chloride cells (CC) and goblet cell (GC). (**b**) Detail of chloride cells (CC) in the lamellar proximal portion. (**c**) Immigrated chloride cells are observed in the apical end of secondary lamellae (black arrowed). Note the thinning of the secondary epithelium (black arrows) and the aneurysms in the lamellar apical tips (asterisks). (**d**) TEM micrograph showing wide intercellular spaces in the main filament (asterisks). Note the reduced and disorganized tubular vesicle system of chloride cells (CC). (**e**) The typical ultrastructural organization of secondary lamellae is completely lost and the thinning of epithelial layers is visible (black arrows). Note the degeneration of pavement cells (PVC) and the appearance of large lacunae (asterisks); n = nucleus, PC = pillar cell. (**f**) Cellular debris, apoptotic bodies, and degenerated nuclei are evident in pavement cells (PVC) and basal cells (BC). Note the secondary epithelium’s detachment from the below basal lamina (asterisks); PC = pillar cell, n = nucleus.

After 48 h of exposure to the high tested concentration, histological examination reveals numerous CCs along the margin of the main filament and the detachment of epithelium from the underlying connective tissue originating large spaces and lacunae. The proliferation of primary epithelium could be seen only at several points, leading to the obliteration of interlamellar space (Figure 3a). CCs are distributed in clusters in the lamellar proximal portion (Figure 3b, and some immigrated CCs (Figure 3c) could also be recognized along the secondary filaments reaching their apical end. It is also possible to observe the appearance of aneurysms (Figure 3c). In the area where the proliferating epithelium does not cover the secondary lamellae, these appear extremely thin, and the epithelial layers are very flattened (Figure 3c). Ultrastructural observations reveal the loose of junctional contact and the presence of wide intercellular spaces (Figure 3d); this phenomenon is particularly evident in CCs, which also show a reduced and disorganizing tubular vesicle system (Figure 3d). Respiratory lamellae lose their typical ultrastructural organization, and the thickness of epithelial layers is greatly reduced; pavement cells show a poor, vacuolated cytoplasm (Figure 3e). Conspicuous cellular debris, apoptotic bodies, and degenerated nuclei are detected in both PVCs and BCs (Figure 3e). In secondary lamellae, the external layer detaches from the basal lamina with the appearance of wide lacunae (Figure 3e,f).

**Figure 4:**
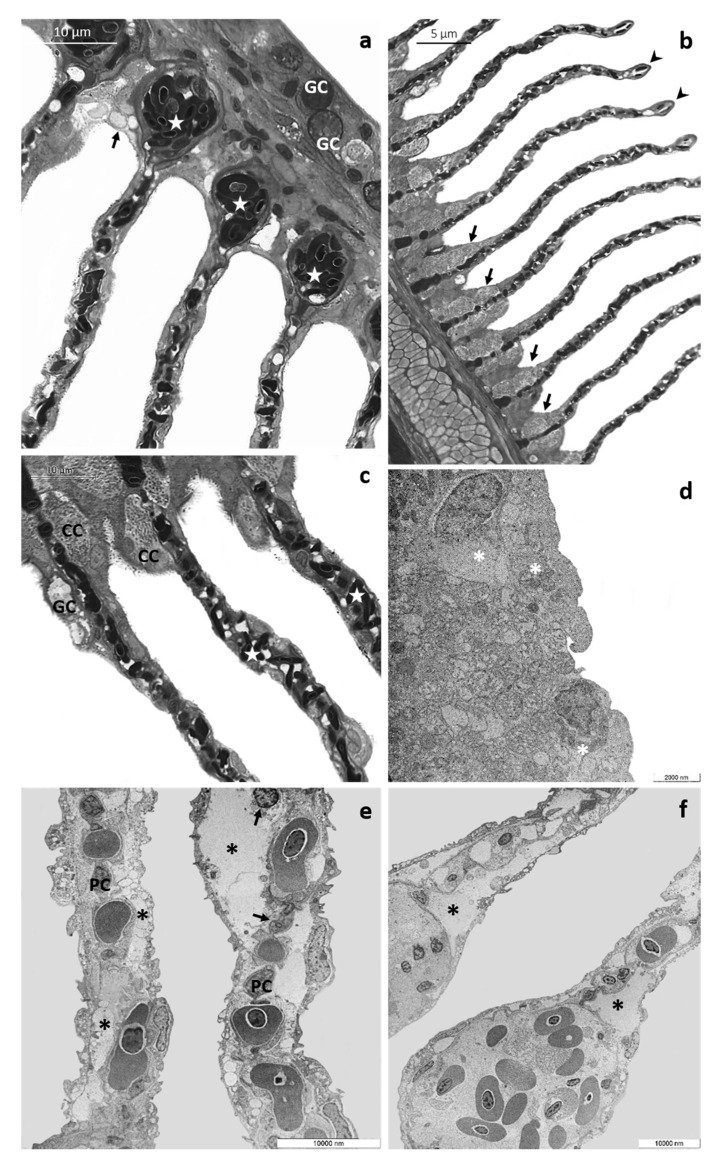
Representative micrographs of *T. pavo* gills after 96 h of exposure to 8 µg/L of CPF. (**a**) Light micrograph showing the flattened main filament and degenerated goblet cells (GC). Note numerous degenerating cells (black arrow) and aneurysms’ appearance in the vascular component (stars). (**b**) The main filament covers the proximal portion of lamellae, and chloride cells increase in number and volume (black arrows). Note also the aneurysms in the lamellar apical tips (black arrowheads). (**c**) Details showing goblet cells (GC) and chloride cells (CC) located in the medial and distal portion of the secondary lamellae; also note the disorganization of vascular component (stars). (**d**) TEM micrograph showing degenerating cells on the main filament (asterisks). (**e**) Secondary lamellae completely lose their typical arrangement, and large lacunae are visible (asterisks). Note the cellular debris (black arrows) and the degeneration of pillar cells (PC). (**f**) Detail of apical tips dilation of secondary lamellae (asterisks).

Alterations involving both primary and secondary filaments further increase after 96 h of exposure to the high tested concentration. Observed under LM, the main filaments appear flattened, and the number of epithelial layers is reduced; it is also possible to recognize strongly modified goblet cells, showing an atypical dark cytoplasm (Figure 4a). Degenerating cells with a scant light cytoplasm are recognizable in the interlamellar region and in the basal portion of secondary lamellae (Figure 4a,b). Moreover, the presence of aneurysms is evident in the vascular component (Figure 4a). The primary epithelium extends from the main filament covering the proximal portion of secondary lamellae (Figure 4b), and it is also possible to recognize some GCs and CCs reaching the medial and distal portion of the secondary lamellae (Figure 4c). Blood congestion with the formation of edema is visible in the distal portion of lamellae (Figure 4b). CCs, both ectopic and in situ, increase in number and volume, and in the vascular compart, the complete disorganization of vascular component is also evident (Figure 4b,c).

Ultrastructural observations clearly show the severe degenerations of the epithelial cells in both primary and secondary filaments (Figure 4d–f). At several points, in the main filament, the epithelial organization in layers is no longer distinguishable, and numerous cells show degenerating organelles and nuclei (Figure 4d). The secondary lamellae completely lose their ultrastructural characteristics, and the lifting of the external layer originates broad areas filled with cellular debris (Figure 4e). The pillar cells often degenerate, and the apical tips are dilated (Figure 4f).

### 3.2. Na^+^/K^+^-ATPase Immunodetection

**Figure 5:**
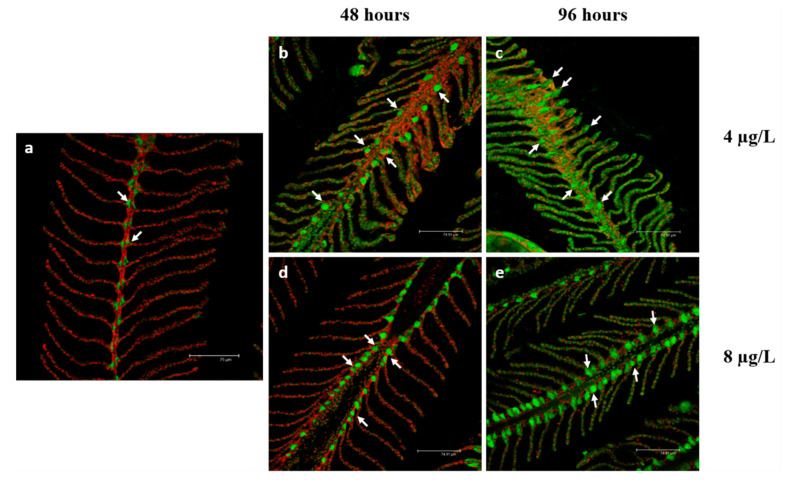
Confocal micrographs of *T. pavo* gill sections labeled with a mouse monoclonal antibody against Na^+^/K^+^-ATPase (green-FITC labeled); nuclei labeled with propidium iodide (red); (**a**) In basal condition, the Na^+^/K^+^-ATPase is located in the CCs of the interlamellar region (white arrows). After 48 (**b**) and 96 h (**c**) of exposure to 4 μg/L of CPF, the Na^+^/K^+^-ATPase immunoreactivity slightly increases compared to the basal condition, and after 96 h of exposure, the signal is also observed in the secondary lamellae (white arrows). After 48 (**d**) and 96 h (**e**) of exposure to 8 µg/L of CPF, the Na^+^/K^+^-ATPase immunoreactivity further increase compared to the basal condition (white arrows). All bar scales 75 µm.

In basal conditions, the immunolabeling for Na^+^/K^+^-ATPase is observed along the filament margins and in the interlamellar region at the level of the CCs (Figure 5a). After 48 h of exposure to the low CPF concentration (Figure 5b), the labeling is detected in the interlamellar region of the main filament remaining localized in the cytoplasm of the chloride cells.

On the contrary, after 96 h of exposure (Figure 5c), the immunosignal is sporadically observed in the secondary lamellae. In both cases, the intensity of Na^+^/K^+^-ATPase slight increase compared to the control group (Table 3 *p* < 0.0001, ***). After exposure to the high concentration of CPF (Figure 5d,e), the labeling is visible in the main filament’s interlamellar region, and the immunosignal intensity further increases compared to the control group (Table 3 *p* < 0.0001, ***).

## 4. Discussion

Over the years, anthropogenic activities, including agriculture, have played a crucial role in determining marine areas’ degradation, possibly giving rise to marine biodiversity loss worldwide. Despite the slower degradation of chlorpyrifos (CPF) in seawater than freshwater, the available data on CPF have mainly focused on freshwater fish, and only two studies have been conducted on gill alterations in juveniles of two coastal and estuarine species after chronic exposure [33,34]. For the first time, we present here the gills’ morpho-functional responses in a marine teleost after short-term exposure to two concentrations of CPF (4 and 8 µg/L). Our findings on *T. pavo*, which document the morphological and ultrastructural alterations of gills induced by CPF, are of ecological interest since they fill a knowledge gap on the effects of this organophosphorus compound on marine organisms.

This study represents basic research on the adverse effects elicited by CPF in *T. pavo* gills. Our results point out relationships between exposure, dose, and response in this organ, and may help to elucidate the mechanisms of the action of OPs compounds.

Among the wide range of outcomes from CPF exposure in fish, including hepatic dysfunction, genotoxicity, hematological alterations, and behavioral disorders, one of the most acknowledged mechanisms by which CPF exerts its toxicity is oxidative stress induction [26,27]. Evidence by biochemical assays indicates that oxidative stress might be involved in CPF toxicity in fish [33,34]. It has been suggested that tissue degeneration observed after CPF exposure in several organs, including gills, may be related to the excessive reactive oxygen species (ROS) by induction of lipid peroxidation and cell structure disruption [26,27,33,34].

### 4.1. Morphological Alterations

We showed that the different doses administered produce a different pathological outcome, and this should especially be considered when implementing a toxicological evaluation (Table 2).

In fact, after exposure to the low-tested concentration, the more frequently detected alteration is an intense proliferation of primary epithelium. A similar response has been observed in several teleost species after exposure to a wide variety of pollutants, including organophosphorus compounds (OPs) [37,40,41,42,43,44], thus suggesting the conservative nature of this trait. In fact, the increase in epithelial thickness allows the gills to mechanically prevent the uptake of unwanted substances. On the contrary, in samples exposed to the high CPF concentration, the primary epithelium proliferation is less extensive, and the most evident alterations are the thinning of secondary lamellae and the ectopia of sporadic chloride cells (CCs) and goblet cells (GCs).

Overall, it seems that exposure to a rather low dose induces a defensive response confirmed by the proliferation of the primary epithelium; however, when the dose of the xenobiotic is above a certain threshold, the epithelium is unable to react with proliferative activity, probably because the energy required by detoxification reduces the mitotic activity of the basal cells and the cell differentiation processes.

In this study, we also demonstrated that aside from pollutant dose, the pathological response is closely linked to the duration of exposure since, for both tested concentrations, the intensity of histological and ultrastructural damages increases over time.

Both vacuolation and degeneration are evident in all experimental groups but their intensity increases over time and is particularly intense after exposure to the highest concentration of CPF. Such processes might be a likely consequence of a direct toxic effect of CPF, and have been observed in seawater (coastal and estuarine species) and freshwater species after both short and long-term exposure to CPF [32,33,34,45].

The distribution of mucous cells in *T. pavo* under basal conditions is limited to the primary epithelium but after CPF exposure we observed an increase and ectopia of GC in all exposed groups becoming more severe with increasing time and dose. Despite both the distribution and mucus composition of GCs diverge among different fish species, comparable effects have also been detected in several freshwater fish after long-term exposure to the same pesticide [27,31]. GCs cells secrete a mucous coat involved in several functions, including contribution to ion exchanges and protection against mechanic injuries, and pollutant intake [27,30]. It has been suggested that the increase in GCs density would be a response to the changes in the ion concentration in the aquatic medium [30]. Thus, it is conceivable that the increased number of GCs observed here would be related to the osmotic distress induced by CPF.

Our observations revealed that pathological modifications also involve gills vascular component inducing the disorganization of the pillar cells (PCs) system, which regulates the blood flow through the capillaries, and, consequently, the dilation of lamellar apical tips and the appearance of aneurysms [46]. These lesions have been detected in all experimental groups, becoming more pronounced with the increase in both the pollutant dose and exposure time. Comparable alterations have been reported after exposure to CPF and other chemicals in both seawater [41,47,48] and freshwater species [30,32,38,46,49]. The loss of the secondary lamellae structural integrity and the lifting of epithelial layers can affect the blood supply to the tissue and, consequently, the osmoregulatory and respiratory functions of the gills.

### 4.2. Na^+^/K^+^-ATPase

In agreement with previous studies, we showed that in control samples, Na^+^/K^+^-ATPase expression is confined at the level of CCs. In contrast, the exposure to the low CPF concentration, inducing an intense ectopia of CCs, also leads to the appearance of the immune signal in the secondary lamellae. Being part of the enzyme pool involved in osmoregulation, Na^+^/K^+^-ATPase may contribute to counterbalance the osmotic disturbance induced by CPF. Therefore, it seems that chloride cells couple the mechanic function with their active enzymatic role, which converge to counteract the noxious effect of CPF. Instead, after exposure to the high CPF concentration, ectopic CCs do not express Na^+^/K^+^-ATPase, and the overall increase in the enzyme activity is exclusively due to the CCs located along the interlamellar region.

It is uncertain whether Na^+^/K^+^-ATPase activity could be directly affected by CPF exposure. Although this may be suggested by a decrease in the expression of this enzyme observed in freshwater fish after exposure to CPF and other pollutants [37,38,50,51,52,53], here we clearly showed a marked increase after exposure to both tested concentrations. No studies are available for comparison with our results, due to the absence of information about Na^+^/K^+^-ATPase modulation in seawater fish after CPF exposure. It should be emphasized that the CCs of marine teleost and freshwater teleost are different in their structure and, in part, in their function [54,55]. It is well established that the principal function of the CCs in marine teleost is osmoregulation, but their physiological role in freshwater fish is still under discussion, and both the mechanism and cell sub-types vary amongst species [54]. More studies are needed to better clarify whether the differences observed in Na^+^/K^+^-ATPase expression would be species–specific or related to different functions of CCs in seawater and freshwater species.

The results obtained, providing evidence of the CPF toxicity on a non-target species, suggest that wild populations can be affected by CPF with profound dramatic consequences on marine fauna and ecosystems.

## Figures and Tables

**Table 1:** Statistic comparison between the groups exposed to CPF and the control group with respect to the presence of morphological alterations in treatment groups (*n* =8).

Fisher’s Exact Probability Tests (Two-Way)			
Group	Effect	No Effect	*p*-Value Summary
Ctrl 48 h	0	8	
Ctrl 96 h	0	8	
Low concentration 48 h	8	0	***
Low concentration 96 h	8	0	***
High concentration 48 h	8	0	***
High concentration 96 h	8	0	***

Asterisks indicate the treated groups that differ from the control using the two-tailed Fisher’s exact probability test (*** *p* < 0.001).

**Table 2:** Semi-quantitative comparison of histological alterations in *T. pavo* gills between the groups exposed to CPF and the control group.

Summary of Histopathological Alterations	Control Group	CPF Exposed Groups			
		Low Concentration		High Concentration	
		48 h	96 h	48 h	96 h
Proliferation of primary epithelium	-	++	+++	+	+
Lifting of secondary epithelium	-	+	++	++	+++
Thinning of epithelial layers	-	-	-	++	+++
Increased number of mucous cells	-	+	++	++	+++
Ectopia of chloride cells and mucous cells	-	+	++	++	+++
Cell degeneration	-	+	++	++	+++
Vascular component alterations	-	+	++	++	+++
Aneurysms’ formation	-	+	++	++	+++

(-) no histological alterations; (+) moderate histopathological alterations; (++) severe histopathological alterations; (+++) very severe histopathological alterations.

**Table 3:** Quantification of Na^+^/K^+^-ATPase protein in the control group and after exposure to CPF.

	Exposure Period	
Group	48 h	96 h
Control	10.71 ± 0.74	10.51 ± 0.35
Low concentration	19.76 ± 0.84 ***	19.40 ± 0.44 ***
High concentration	20.12 ± 0.58 ***	19.95 ± 0.69 ***

The values indicate the percentage of the section’s area expressing fluorescent signal –SEM (*n* = 8). Asterisks indicate the treated groups that differ from the control, *** *p* < 0.0001.
